# Supplementary material for: The Daily Mile: Whole-school recommendations for implementation and sustainability. A mixed-methods study
Source: PLoS One. 2020 Feb 5;15(2):e0228149. doi: 10.1371/journal.pone.0228149 (PMC7001902; doi:10.1371/journal.pone.0228149)
Supplement: S1 Appendix — Schematic diagram of data collection time points (baseline and follow up)–Schools A-F. (DOCX) [file pone.0228149.s001.docx]

S1 Appendix: Schematic diagram of data collection time points (baseline and follow up) – Schools A-F
