# Supplementary material for: The Daily Mile: Whole-school recommendations for implementation and sustainability. A mixed-methods study
Source: PLoS One. 2020 Feb 5;15(2):e0228149. doi: 10.1371/journal.pone.0228149 (PMC7001902; doi:10.1371/journal.pone.0228149)
Supplement: S2 Appendix — (DOCX) [file pone.0228149.s002.docx]

| Transcript Number | School | Participant | Timepoint |
| --- | --- | --- | --- |
| 1 | D | Pupil Focus Group | Baseline |
| 2 | D | Pupil Focus Group | Baseline |
| 3 | D | Headteacher Interview | Follow up |
| 4 | D | Teacher Interview | Follow up |
| 5 | B | Teacher Interview | Baseline |
| 6 | B | Teacher Interview | Follow up |
| 7 | B | Teacher Interview | Baseline |
| 8 | B | Teacher Interview | Follow up |
| 9 | B | Teacher Interview | Baseline |
| 10 | B | Teacher Interview | Baseline |
| 11 | C | Teacher Interview | Follow up |
| 12 | A | Teacher Interview | Follow up |
| 13 | E | Pupil Focus Group | Follow up |
| 14 | E | Pupil Focus Group | Follow up |
| 15 | F | Headteacher Interview | Follow up |
| 16 | F | Pupil Focus Group | Follow up |
| 17 | F | Pupil Focus Group | Follow up |

S2 Appendix: Interview and Focus Group Participation
