# Supplementary material for: The Daily Mile: Whole-school recommendations for implementation and sustainability. A mixed-methods study
Source: PLoS One. 2020 Feb 5;15(2):e0228149. doi: 10.1371/journal.pone.0228149 (PMC7001902; doi:10.1371/journal.pone.0228149)
Supplement: S3 Appendix — (DOCX) [file pone.0228149.s003.docx]

**Focus Group Topic Guide (Pupils)**

**Baseline**

Brief introduction

1. Can you tell me what you thought about the daily mile when you first heard about it?
2. Do you think you will enjoy the daily mile? Will your classmates enjoy the daily mile?
3. What time of day and where will you do the daily mile? Do you think this is the best time of day and the best place to do it?
4. Do you think the daily mile will make any difference to how you and the people in your class learn or behave? In what way?
5. Do you think the daily mile will change how well you are doing in school and in what way?
6. Can you tell me if you think there will be any problems with the daily mile? Do you have any suggestions of how to make it work better?
7. Do you want to do the Daily Mile? Prompts: Does everyone in your class want to do the daily mile?
8. Do you have anything else to add about the daily mile?

**Focus Group Topic Guide (Pupils)**

**Follow-up**

Brief introduction

1. Can you tell me what you thought about the daily mile when you first heard about it? And what do you think about it now? Prompt: What is good/bad?
2. Do you enjoy the daily mile? Do your classmates enjoy the daily mile?
3. What time of day and where do you do the daily mile? Do you think this is the best time of day and the best place to do it? Prompt: Have there been any times when you haven’t been able to do the daily mile and what were the reasons? What did you do instead?
4. Do you think the daily mile is making any difference to how you and the people in your class learn or behave? In what way? Is it making any difference to your concentration?
5. Do you think the daily mile is changing how well you are doing in school and in what way?
6. Can you tell me if there are any problems with the daily mile? Do you have any suggestions of how to make it work better?
7. Would you like to carry on with the daily mile and why? Prompts: Does everyone in your class want to carry on with the daily mile?
8. Do you have anything else to add about the daily mile?

**Interview Topic Guide (Headteacher)**

**Follow-up**

Brief introduction

1. Can you tell me about how the daily mile has gone?
2. What was your motivation for starting the daily mile in your school?
3. How did you feel about the implementation of the daily mile? Barriers/challenges. Were there any issues? Were all staff happy with the implementation?
4. How did your staff/pupils feel about taking part in the daily mile?
5. Can you tell me whether you think the daily mile made any difference to how your pupils learnt or behaved? Or any other whole school effects?
6. Do you think there were any problems with the daily mile? Do you have any suggestions of how to make it work better if other schools were to deliver the project?
7. Would you like to continue the daily mile? Do you think other schools should deliver the daily mile? Why/why not?
8. Do you have anything else to add about your thoughts about this project in general?

**Interview Topic Guide (Teacher)**

**Baseline**

Brief introduction

1. What do you know about the daily mile? Prompt: Have your pupils done the daily mile or anything similar in the past?
2. What were your initial thoughts of the Daily Mile?
3. How do you feel about delivering/implementing the daily mile to your class and why?
4. How is the daily mile being implemented within the school? Prompt: Are all staff onboard? Any issues?
5. Can you tell me whether you think the daily mile will make any difference to how pupils learn or behave? Or any other effects?
6. Can you tell me whether you think the daily mile will change how well your pupils do in school and in what way?
7. Are there currently any problems with the daily mile? Do you have any suggestions of how to make it work well?
8. How do your pupils feel about the daily mile? Prompts: What do they like/dislike?
9. Do you have anything else to add about your thoughts about this project in general?

**Interview Topic Guide (Teacher)**

**Follow-up**

Brief introduction

1. Can you tell me about how the daily mile has gone?
2. How did you feel about the implementation of the daily mile? Were there any issues? Were all staff happy with the implementation?
3. Did you feel supported throughout the delivery of the project? Did you receive any training to deliver it?
4. Can you tell me whether you think the daily mile made any difference to how your pupils learnt or behaved? Or any other effects?
5. Can you tell me whether you think the daily mile changed how well your pupils were doing in school and in what way?
6. Do you think there were any problems with the daily mile? Do you have any suggestions of how to make it work better if other schools were to deliver the project?
7. How did your pupils feel about taking part in the daily mile?
8. Would you like to continue the daily mile? Do you think other schools should deliver the daily mile? Why/why not?
9. Do you have anything else to add about your thoughts about this project in general?
