# Supplementary material for: The Daily Mile: Whole-school recommendations for implementation and sustainability. A mixed-methods study
Source: PLoS One. 2020 Feb 5;15(2):e0228149. doi: 10.1371/journal.pone.0228149 (PMC7001902; doi:10.1371/journal.pone.0228149)
Supplement: S4 Appendix — (DOCX) [file pone.0228149.s004.docx]

| **Theme** | **Sub-theme** |
| --- | --- |
| The Daily Mile implementation | Flexible vs rigid principles |
|  | Curriculum time vs playtime |
|  | Competitive vs non-competitive |
|  | Active teachers vs passive teachers |
|  | Supported vs unsupported |
|  | Summer vs winter |
| Impact on learning, health and wellbeing | Behaviour and concentration |
|  | Physical activity and sport |
|  | Psychological benefits |
|  | Social benefits |
