# Supplementary material for: The Daily Mile: Whole-school recommendations for implementation and sustainability. A mixed-methods study
Source: PLoS One. 2020 Feb 5;15(2):e0228149. doi: 10.1371/journal.pone.0228149 (PMC7001902; doi:10.1371/journal.pone.0228149)
Supplement: S5 Appendix — Descriptive characteristics of shuttles and CRF by school (A-F). (DOCX) [file pone.0228149.s005.docx]

|  | School A | School B | School C | School D | School E | School F |
| --- | --- | --- | --- | --- | --- | --- |
| Difference in shuttles (baseline – follow-up) | 1.4 ± 12.8 (38)  (95% CI: -2.8 to 5.6) | 1.5 ± 6.9 (26)  (95% CI: -1.3 to 4.3) | 8.1 ± 13.8 (29)  (95% CI: 2.9 to 13.3) | 7.9 ± 9.0 (46)  (95% CI: 5.2 to 10.6) | 2.8 ± 17.5 (41)  (95% CI: -2.7 to 8.3) | 12.6 ± 8.7 (23)  (95% CI: 8.8 to 16.4) |
| Difference in shuttles imputed (baseline – follow | 2.0 ± 12.6 (42)  (95% CI: -1.9 to 5.9) | 4.8 ± 11.6 (39)  (95% CI: 1.0 to 8.6) | 5.2 ± 14.7 (39)  (95% CI: 0.4 to 10.0) | 7.3 ± 12.6 (51)  (95% CI: 3.8 to 10.8) | 1.8 ± 19.3 (60)  (95% CI: -3.2 to 6.8) | 10.4 ± 15.2 (31)  (95% CI: 4.8 to 16.0) |
| Baseline shuttles | 43.2 ± 20.7 (42) | 26.2 ± 17.3 (33) | 14.9 ± 10.4 (29) | 28.8 ± 16.6 (49) | 36.1 ± 19.1 (47) | 28.7 ± 18.0 (29) |
| Follow up shuttles | 45.4 ± 22.4 (38) | 29.6 ± 20.1 (32) | 23.5 ± 12.8 (39) | 36.4 ± 18.3 (47) | 37.6 ± 20.1 (53) | 41.2 ± 22.0 (25) |
| Baseline shuttles imputed | 43.2 ± 20.7 (42) | 25.0 ± 16.4 (39) | 18.2 ± 11.4 (39) | 29.4 ± 16.5 (51) | 36.3 ± 17.5 (60) | 29.7 ± 17.5 (31) |
| Follow up shuttles imputed | 45.3 ± 21.5 (42) | 29.8 ± 19.0 (39) | 23.5 ± 12.8 (39) | 36.7 ± 18.0 (51) | 38.1 ± 19.4 (60) | 40.1 ± 20.5 (31) |
| Fit (baseline) | 79% (33) | 40% (13) | 7% (2) | 39% (19) | 64% (30) | 48% (13) |
| Fit (follow up) | 76% (29) | 41% (13) | 32% (12) | 68% (32) | 58% (31) | 75% (18) |
| Fit imputed (baseline) | 79% (33) | 33% (13) | 13% (5) | 41% (21) | 67% (40) | 53% (16) |
| Fit imputed (follow up) | 79% (33) | 44% (17) | 32% (12) | 69% (35) | 63% (38) | 73% (22) |

*Mean ± SD (n); 95% CI = 95% confidence interval, % (n)*
