# Supplementary material for: The Daily Mile: Whole-school recommendations for implementation and sustainability. A mixed-methods study
Source: PLoS One. 2020 Feb 5;15(2):e0228149. doi: 10.1371/journal.pone.0228149 (PMC7001902; doi:10.1371/journal.pone.0228149)
Supplement: Attachment — Submitted filename: Response to Reviewers.docx [file pone.0228149.s007.docx]

Emily Marchant

Medical School, Swansea University

[E.K.Marchant@swansea.ac.uk](mailto:E.K.Marchant@swansea.ac.uk)

2nd^th^ January 2020

Re: Response to reviewers [PONE-D-19-21022R1]

Dear Dr Visram,

Thank you for the opportunity to further revise our manuscript, *The Daily Mile: whole-school recommendations for implementation and sustainability. A mixed-methods study.* On behalf of the co-authors, I would like to thank the reviewers again for providing their valued input and feedback on the manuscript. I would also like to thank the reviewers for their positive comments regarding the revised manuscript and its improvement following the first round of reviews. We believe we have further addressed their queries and that their suggestions have significantly strengthened the manuscript.

All changes addressing reviewers’ comments are highlighted as track changes in the attachment ‘Revised Manuscript with Track Changes’. Furthermore, an unmarked manuscript is attached ‘Manuscript’. Please find response to the reviewers’ comments below and associated changes with reference to page numbers.

Thank you again for the opportunity to revise the manuscript. I look forward to hearing from you.

Yours sincerely,

Emily Marchant

**Reviewer #1**

**Thank you for your comprehensive response to the reviewers comments. I am satisfied you have addressed my comments, and I feel the manuscript is improved as a result. There are just a few minor amends to address.**

**Introduction - line 65 - 'the' missing from the sentence before 'latest'?**

Response

Thank you for bringing this oversight to my attention. I have addressed this as outlined below.

Change

Page 3: Furthermore, survey level data from the latest Active Healthy Kids Wales Report Card within Wales, United Kingdom suggests that just 34% of children aged 3-17 years are meeting these guidelines[9]

**Quantitative results p 31 line 744 - please could you insert the percentages for number of pupils in brackets**
Response

I agree that displaying the percentage of eligible pupils that participated in the measures would provide further clarity to the reader. I have updated this within the manuscript.

Change

Page 30: There was a total of 336 pupils in years 5 and 6 attending the six primary schools in this study. From this sample of eligible pupils, 229 pupils (68%) participated in the 20m SRT at baseline and 235 pupils (70%) at follow up. In total, 204 pupils (61%) completed the 20m SRT at both time points.

**Discussion - line 785-787 - Please review this sentence, currently it doesn't make sense**

Response

Thank you for suggesting a restructure of this sentence, I agree that it was not clear I have addressed your concerns with the following change:

Change

Page 34: Removed original sentence

Page 34: After the initial excitement of a new school activity, pupils commented on feeling bored and lacking enjoyment, impacting on pupils’ participation and the longer-term sustainability of TDM

**Strengths and limitations - line 1005 amend 'adopted' to 'adoption'**

Response

Thank you again for raising this oversight. I have made the amendment as instructed.

Change

Page 40: In this research study, a natural experimental approach was chosen due to the widespread adoption of TDM as a result of political and media support.

**Reviewer #2**

**I would firstly like to congratulate the authors on their responses and revision of this paper. The manuscript is much improved and the authors should be commended on their drive to provide transparent and rigorous science on such a politically charged intervention. I have two remaining queries that I would like the authors to address.**

Response

**The first is with regards to the presentation of the CRF data. I am glad the authors have now refocused their work away from this outcome. For further transparency, I would like the authors to consider adding 95% confidence intervals to their CRF changes, as this will further highlight the wide variability in the change in CRF scores and allows the reader to easily see this. Once this change has been made in the manuscript, I also request that this information is included in the abstract and if word count allows, a few words added to highlight the variability in the data and the implications of this.**
Response

Thank you for your suggestion of adding in 95% confidence intervals to the CRF changes data. I agree that this would provide clarity on the variability within the data. I have included the 95% confidence intervals within the manuscript and have highlighted the wide variability within the results and discussion section. As the abstract is already at the word limit, I am unable to include this information in the abstract.

Change

Page 31: However, these results exhibit large standard deviation and wide 95% confidence intervals (deprived: 2.0 to 7.4, non-deprived: 2.3 to 7.3), demonstrating the variability that is present among this sample

Page 32: Table 2 updated with confidence intervals

Page 38-39: An equal increase in the number of shuttles run by the deprived and non-deprived groups was observed, however, the wide confidence intervals present within this data demonstrate the variability of changes in children’s CRF.

**My second question relates to how the focus group participants were chosen. Am I correct in** **thinking the class teachers chose the school pupils based on the list of those who provided consent? If so, could this be viewed as 'cherry picking', in that the teachers may have chosen pupils based on their knowledge of them (e.g. choosing pupils that they thought enjoyed the programme/ were likely to talk positively about it/ were already 'good standing' pupils within the school community). I would like the authors to consider this point and add this potential bias to the limitations of the study**

Response

Thank you for requesting further information on the sampling for focus groups within this study. I agree that this information is important for the reader. I understand your concerns regarding the potential for ‘cherry picking’, however, teachers were reminded of the importance of selecting children that fulfilled the criteria (e.g. mixed physical activity ability). Having worked closely with the schools and teachers within this study, the authors have confidence that this was achieved. To address your queries, I have explained this further in the results section and in the strengths and limitations section as outlined below.

Change

Page 10: Teachers were reminded of the need to include pupils of a range of physical activity abilities.

Page 41: Focus group sampling was achieved through teachers selecting consented pupils fulfilling a criteria of mixed gender and physical activity abilities. Although teachers were reminded of the importance of selecting a variety of pupils with a range of abilities in order to capture a variety of experiences, there is potential that this method could cause bias in results through preferential selection.
